# Digital intelligence in dental technology education: curriculum and practical teaching reform

**DOI:** 10.3389/fmed.2026.1912754

**Published:** 2026-08-27

**Authors:** Zhi-Fa Wang, Yao-Xiang Xu, Hao-Ran Zhao, Wen-Lin Xiao

**Affiliations:** 1 School of Medicine, Qingdao Huanghai University, Qingdao, Shandong, China; 2 Department of Oral and Maxillofacial Surgery, Affiliated Hospital of Qingdao University, Qingdao, Shandong, China; 3 School of Stomatology, Qingdao University, Qingdao, Shandong, China

**Keywords:** dental technology education, digital intelligence, interdisciplinary integration, large language models, practical teaching reform, virtual simulation

## Abstract

Oral healthcare is rapidly progressing from basic digitalization toward increasingly AI-enabled and data-driven practice, reshaping the competencies required of dental technology professionals and challenging traditionally technician-oriented educational models. This narrative review examines how digital intelligence can support curriculum development and practical teaching reform in dental technology education, with particular attention to application-oriented undergraduate universities. Drawing on representative literature related to digital workflows, virtual simulation, haptic systems, three-dimensional printing, large language models, generative artificial intelligence (AI), blended learning, and interprofessional and industry–education collaboration, this review critically synthesizes current evidence and proposes a literature-informed integrative conceptual framework for competency-based curriculum planning. Available evidence suggests that digital intelligence may broaden educational objectives beyond manual technical skills to include digital design, data interpretation, intelligent decision support, ethical awareness, and interdisciplinary communication. These technologies may also support a transition from conventional repetitive training toward more progressive combinations of virtual simulation, physical operation, chairside observation, project-based learning, and workplace-oriented internship. However, important controversies and challenges remain. Artificial intelligence and virtual simulation should remain supervised complements rather than replacements for teachers, conventional laboratory training, or clinical experience. AI-generated outputs may be affected by hallucination, algorithmic bias, limited explainability and reproducibility, and student overdependence, thereby requiring faculty validation, human oversight, and clear governance under evolving regulatory conditions. Current research gaps include limited dental technology-specific evidence, insufficient long-term evaluation of competency development and workplace transferability, and persistent barriers related to equipment investment, software access, faculty training, and curriculum alignment. Future reform should therefore move from tool-centered adoption toward competency-centered curriculum reconstruction, emphasizing progressive virtual–physical–clinical integration, personalized assessment, faculty development, medicine–engineering collaboration, and sustainable industry–education synergy. Overall, digital intelligence-driven reform may help align dental technology education with changing practice needs; however, its sustained educational benefits remain uncertain because current evidence is derived mainly from pilot studies, cross-sectional surveys, short-term simulations, and review-based synthesis.

## 1 Introduction

Entering the third decade of the 21st century, oral healthcare is undergoing a continuing transition from basic digitalization toward increasingly AI-enabled and data-driven practice (1). From multimodal imaging integration based on cone-beam computed tomography (CBCT) and precise three-dimensional data acquisition through intraoral scanning (IOS), to the clinical implementation of computer-aided design and computer-aided manufacturing (CAD/CAM) and three-dimensional (3D) printing, a complete digital workflow is progressively reshaping the diagnostic, treatment planning, and prosthesis design–manufacturing chain in dentistry (2). These technology-driven changes in the dental industry have also generated new competency demands for higher dental education, particularly for dental technology programs, highlighting the need for a more systematic integration of digital skills training to meet the requirements of future clinical and technical practice (3). In this review, “digital intelligence” is used as an umbrella term encompassing digital workflows, data-driven systems, virtual simulation, and AI-enabled educational technologies. “Artificial intelligence” refers specifically to algorithmic systems, including generative AI and large language models.

For a long time, the education of dental technology professionals in China has remained largely grounded in traditional technician-oriented training, and the associated educational system and curriculum development have long been in a state of exploration and transition (4). In the face of highly automated modern dental laboratory centers and digital oral healthcare systems, training pathways that still emphasize gypsum model analysis, manual wax-up carving, and conventional investing and casting procedures have gradually shown a growing mismatch with ongoing industry digitalization. Recent studies have shown that digital transformation is reshaping denture fabrication and prosthodontic workflows, while substantially increasing the demand for multidisciplinary professionals with digital competencies (5). As a result, graduates entering positions related to digital design, surgical guide planning, and intelligent prosthesis production often still require additional workplace adaptation or supplementary training to bridge the competency gap, which to some extent reflects a structural mismatch between curricular provision and occupational needs (6). Meanwhile, considerable disparities remain among Chinese undergraduate dental schools in the adoption of digital and intelligent teaching resources such as virtual simulation. A nationwide survey reported that the overall implementation rate of such courses remains relatively low, with broader adoption in “Double First-Class” universities, whereas ordinary institutions are more constrained by limited teaching resources and curriculum development capacity (7).

In recent years, dental education reform, both in China and internationally, has increasingly shifted toward competency-based training and the integration of digital technologies (8). Systematic reviews suggest that digital simulation technologies, including virtual reality, may improve selected short-term outcomes such as knowledge acquisition, operative performance, confidence, and psychomotor skills. However, heterogeneity among studies and limited follow-up restrict conclusions regarding sustained competency development and transfer to authentic practice (9). However, these technologies are more appropriately regarded as important complements to conventional practical teaching rather than complete replacements (9). At the same time, artificial intelligence (AI) has been progressively incorporated into multiple areas of dental education, including learning support, diagnostic training, assessment and feedback, and instructional content generation, indicating that digital intelligence-enabled education is becoming an important direction for innovation in dental training (10). In addition, interprofessional education has been incorporated into undergraduate dental curricula in many countries, most commonly in collaboration with medicine, nursing, and pharmacy, and has been shown to promote core competencies such as role recognition, teamwork, and communication (11). Nevertheless, how to translate these emerging educational advances into reproducible, practical, and implementable teaching strategies for application-oriented undergraduate institutions, which often face relative resource constraints and place strong emphasis on hands-on competence, remains an important theoretical and practical challenge.

Against this background, the present narrative review examines curriculum development and practical teaching reform in dental technology education supported by digital intelligence. The review aims to critically synthesize evidence concerning digital workflows, artificial intelligence, virtual simulation, three-dimensional printing, blended learning, interprofessional collaboration, and industry–education integration; identify major implementation barriers and research gaps; and propose a literature-informed integrative conceptual framework for application-oriented dental technology programs.

**Figure 1:**
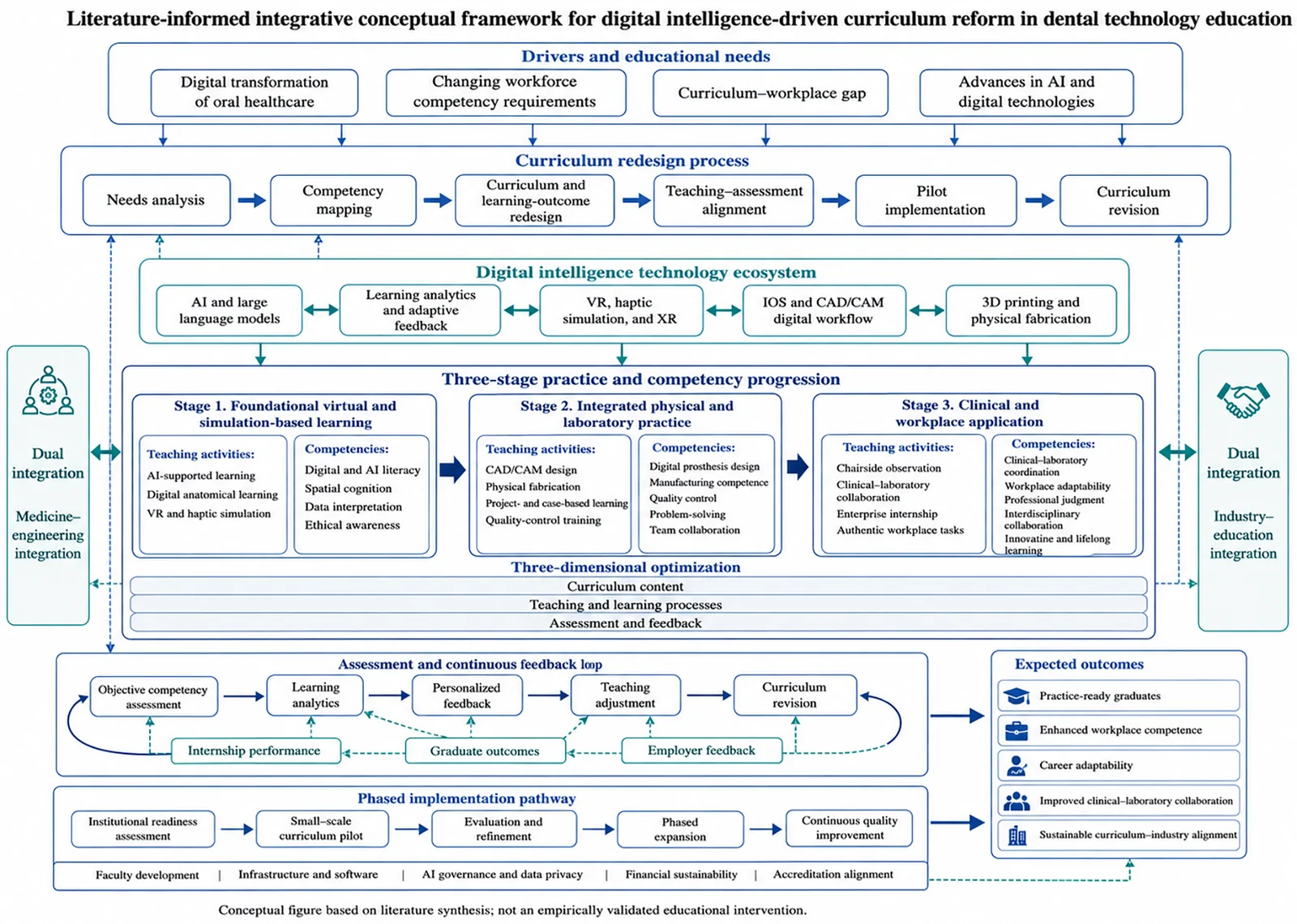
Literature-informed integrative conceptual framework for digital intelligence-driven curriculum reform in dental technology education. The framework was newly synthesized by the authors from existing literature on competency-based education, digital learning, simulation-based training, interprofessional education, and industry–education collaboration. Curriculum redesign begins with needs analysis and competency mapping and is supported by an interconnected digital technology ecosystem, including artificial intelligence and large language models, learning analytics, virtual reality and haptic simulation, extended reality, intraoral scanning, computer-aided design and manufacturing, and three-dimensional printing. Competency development progresses from foundational virtual and simulation-based learning to integrated laboratory practice and clinical or workplace application. Curriculum content, teaching and learning processes, and assessment and feedback are optimized across all three stages, while medicine–engineering and industry–education integration provide cross-cutting support. Assessment data, internship performance, graduate outcomes, and employer feedback are returned to curriculum planning through a continuous feedback loop. The framework is intended as a conceptual guide and has not yet been empirically validated as an integrated educational intervention.

The proposed framework, termed “three-stage practice, three-dimensional optimization, and dual integration,” was synthesized from existing literature on competency-based education, digital learning, simulation-based training, interprofessional collaboration, and industry–education integration. It is intended as a context-specific guide for curriculum planning rather than as a new educational theory or an empirically validated intervention. The methods used to identify, screen, and select the literature are described below. Figure 1 presents the literature-informed conceptual framework proposed by the authors for digital intelligence-driven curriculum reform in dental technology education; it does not represent the literature-search or study-selection process of this narrative review.

## 2 Methods of the narrative review

### 2.1 Search strategy

A structured narrative literature search was conducted in PubMed/MEDLINE, Web of Science Core Collection, Scopus, and the Education Resources Information Center (ERIC) for English-language articles published between 1 January 2010 and 15 July 2026. Priority was given to evidence published during the most recent decade, while earlier foundational studies were retained when relevant. Google Scholar and citation tracking were used to identify additional publications.

The principal search strategy combined terms related to dental education (“dental education,” “dental technology education,” “dental technician education,” or “dental laboratory technology education”) with terms related to digital and educational innovation, including “digital dentistry,”“artificial intelligence,” “generative artificial intelligence,” “large language model*,” ChatGPT, “retrieval-augmented generation,” “virtual reality,” haptic*, “extended reality,” “augmented reality,”“three-dimensional printing,” CAD/CAM, “intraoral scan*,” “digital workflow*,” “blended learning,” “interprofessional education,” and “curriculum reform.” Search terms were adapted to the indexing requirements of each database.

### 2.2 Eligibility and study selection

Peer-reviewed original studies, surveys, qualitative and mixed-methods studies, reviews, consensus statements, perspective articles, and educational practice reports were eligible when they addressed digital competence, artificial intelligence-assisted education, simulation technologies, three-dimensional printing, digital workflows, blended learning, interprofessional collaboration, curriculum development, or institutional governance in dental or dental technology education.

Studies from medical or other health-professions education were included only when their educational concepts were directly applicable to dental technology education. Duplicates, non-English publications, conference abstracts without full texts, theses, non-peer-reviewed materials, and studies without clear educational or curricular relevance were excluded.

After duplicate removal, one author screened titles, abstracts, and potentially eligible full texts. A second author reviewed and confirmed the final selection, and disagreements were resolved through discussion.

### 2.3 Evidence selection and synthesis

Because this was a narrative review, the aim was not to identify every eligible publication. Representative literature was selected according to relevance to dental technology education, methodological rigor, recency, relevance to curriculum or competency development, diversity of technologies and educational settings, and contribution to the proposed conceptual framework.

Information on study design, participants, educational intervention, outcomes, and limitations was synthesized thematically. Quantitative pooling and formal risk-of-bias assessment were not performed because of substantial methodological heterogeneity. Evidence-strength categories in Tables 1, 2 were descriptive rather than formal GRADE assessments.

**Table 1:** Critical comparison of representative AI studies in dental education.

Study	Design/sample	Educational outcome	Evidence	Main limitation
Kavadella et al. (26)	Mixed-method comparison; *n* = 77	Better short-term knowledge; useful learning support	Moderate	Single center; short follow-up
Lin et al. (29)	Pre–post comparison; *n* = 64	Better landmark accuracy; smaller learner differences	Moderate	Task-specific; no long-term data
Busch et al. (33)	International survey; *n* = 4596, including 205 dental students	High demand for AI teaching; low preparedness	Low	Self-reported; small dental subgroup
Hassanein et al. (58)	Retrospective validation; *n* = 262; 2358 responses	Model agreement varied; errors could be over-scored	Moderate	Single course; retrospective design

AI, artificial intelligence. Evidence strength was judged descriptively from design, sample, outcome objectivity, and follow-up; it was not a formal GRADE assessment.

**Table 2:** Critical comparison of representative digital simulation studies.

Study	Design/sample	Educational outcome	Evidence	Main limitation
Farag and Hashem (39)	Single-arm pre–post; *n* = 21	Better preparation scores; shorter time	Low	Small sample; no control
Duan et al. (40)	Within-student comparison; *n* = 98	Supported combined VR, plastic, and 3D training	Moderate–low	Short-term; partly perception-based
Li et al. (44)	Randomized trial; *n* = 80	VR and phantom-head outcomes were similar	Higher	One task; small groups
Liu et al. (48)	Prospective cohort; *n* = 109	Better RPD design at 1 month and 1 year	Moderate	Nonrandomized; no workplace outcome

VR, virtual reality; 3D, three-dimensional; RPD, removable partial denture. Evidence strength was descriptive and was not a formal GRADE assessment.

## 3 Top-level design and theoretical framework for digital intelligence-enabled dental education

Before discussing specific curriculum restructuring strategies and technological applications, it is necessary to clarify, from a broader perspective, the theoretical basis, core frameworks, and key issues associated with embedding digital intelligence technologies into medical education, including professionalism, equity, and implementation pathways. The digital transformation of education is not merely a matter of replacing the traditional blackboard with electronic screens; rather, it requires the reconstruction of curriculum objectives, learning outcomes, and instructional approaches around digital health competencies, thereby promoting a shift in medical education from a knowledge-transmission model toward one that places greater emphasis on learner development and professional competency cultivation (12).

### 3.1 Construction of a core competency framework for digital education

International reviews and perspective studies indicate that the digital competency framework in dental education is no longer limited to the operation of individual software programs or devices. Instead, it has gradually expanded into a curricular system encompassing intraoral scanning, digital imaging, three-dimensional imaging, CAD/CAM, and related information technologies, while also requiring parallel reforms in teaching content, pedagogy, faculty development, and infrastructure provision (13). Within this framework, curriculum standards in dentistry and dental technology should move beyond isolated tool-based instruction toward core competency training organized around the digital workflow. A survey of North American dental schools showed that although CAD/CAM and digital scanning have been incorporated into undergraduate education in most institutions, substantial differences remain in the depth of curricular integration, frequency of use, and resource allocation across schools (14).

This top-level design implies that universities should not only train students to operate software and equipment, but also cultivate critical thinking, the ability to judge the boundaries of algorithm applicability, and awareness of the risks of AI misuse and bias in clinical decision-making (15). Practice in dental education in China further suggests that the combined use of AI courses, knowledge graphs, and online virtual simulation platforms is shifting education from a credit-oriented model to a competency-oriented one. Through learning-process tracking, personalized pathway planning, and real-time feedback, these approaches more closely connect curriculum development, competency cultivation, and educational assessment (16).

### 3.2 Ethical risks and governance challenges in intelligent educational environments

As large language models (LLMs) and other forms of generative artificial intelligence are increasingly introduced into dental education, their potential benefits must be considered alongside substantial educational and professional risks. AI systems may generate hallucinated facts, fabricated references, or inappropriate recommendations in fluent and convincing language, making such errors difficult for inexperienced learners to identify. Algorithmic bias in training data may also produce unequal or contextually inappropriate outputs, while limited explainability can prevent students and educators from understanding how conclusions were generated or whether they are professionally defensible (17, 18).

Reproducibility is another important limitation because AI outputs may vary according to model version, software updates, prompt wording, reference sources, and generation settings. Consequently, apparently identical educational tasks may produce different results across users, institutions, or time points. Excessive reliance on such tools may also encourage students to outsource information retrieval and reasoning before establishing sufficient foundational knowledge, thereby weakening critical appraisal and independent professional judgment (18, 19).

AI-generated teaching materials, case scenarios, feedback, and assessment results should therefore be reviewed and validated by qualified faculty before educational use. Students should be trained to use AI critically and safely, while teachers retain responsibility for evaluating the professional accuracy and educational appropriateness of AI-generated outputs (20). Institutions should also require disclosure of AI use, verification against authoritative sources, and clear assignment of responsibility for inaccurate or harmful outputs (21). Because regulation of generative AI in dental education remains incomplete and continues to evolve, institutional policies should be regularly reviewed in relation to patient safety, data protection, academic integrity, professional accountability, and accreditation requirements (22).

## 4 Multidimensional integration of large language models and generative AI in curriculum reform

Since late 2022, LLMs represented by ChatGPT have rapidly advanced in natural language understanding and generation, and their potential applications in dental education have quickly become a major research focus. Recent reviews have further shown that large language models, large vision models, and multimodal models are expanding the application of AI across dental diagnosis, clinical documentation, treatment planning, and multiple dental specialties, providing a broader technological basis for their incorporation into dental education (23). In dentistry and its related technical fields, generative AI is no longer viewed merely as a passive tool for information retrieval. Emerging studies suggest that it can also be used to generate teaching content, provide personalized feedback, construct case-based scenarios, and support instructional interaction as well as learning-process optimization, thereby offering new technological pathways for curriculum reform (19).

### 4.1 Intelligent agent applications driving innovation in specialty course models

AI-based chatbots and virtual patients have been used to support history taking, communication, case discussion, and early clinical reasoning. A dual-chatbot activity simulating patient interviews and supervisory discussion was considered relevant to authentic dental scenarios and helped students identify weaknesses in history taking (24). A separate pilot study also reported favorable student acceptance of chatbot-based virtual patient interactions for diagnostic and communication training (25).

In broader learning tasks, ChatGPT-assisted activities were associated with better short-term knowledge performance than conventional literature searching, although students also reported overgeneralized responses and fabricated citations (26). AI-standardized patients have similarly been explored for case-management and crisis-training exercises without involving real patients (27).

Nevertheless, the current evidence remains preliminary and is derived mainly from single-setting studies assessing feasibility, short-term knowledge, satisfaction, or self-reported confidence rather than sustained professional competence. Differences in model version, prompting strategy, educational scenario, and faculty supervision also limit comparison across studies; future research should therefore report model and prompt details, reference sources, and faculty-validation procedures (19). Because these studies involved dental or medical learners rather than dental technology students, their findings should not be directly extrapolated to laboratory-based competencies such as digital prosthesis design, manufacturing quality control, and clinical–laboratory communication. AI-based virtual patients should therefore be regarded as complementary teaching tools pending longitudinal evaluation of competency development and workplace performance.

### 4.2 Intelligent assessment and instructional support across dental specialties

The integration of AI and generative models into different areas of dental education is gradually shifting student assessment from coarse end-point scoring toward a formative model characterized by process tracking, immediate feedback, and specialty-specific knowledge enhancement. In endodontic education, retrieval-augmented generation (RAG)-based LLMs that integrate textbooks, clinical guidelines, and multimodal teaching resources into local knowledge bases can significantly improve performance in terms of clinical accuracy, relevance, completeness, and professional judgment, thereby supporting real-time feedback and formative assessment in specialty teaching (28). In orthodontics, digital and AI-assisted cephalometric training can markedly improve students' landmark identification accuracy through real-time feedback and visual error-correction mechanisms, while also helping reduce performance disparities among learners with different levels of clinical experience (29). In oral and maxillofacial radiology, AI-driven adaptive learning, automated assessment, and clinical decision-support tools are promoting a shift in image interpretation training from static knowledge delivery to a more dynamic, interactive, and competency-oriented approach (30). Clinical AI evidence also requires cautious interpretation. A systematic review of facial fracture detection studies reported promising diagnostic performance but identified predominantly retrospective single-center designs, limited external validation, and insufficient explainability, highlighting the need to teach critical evaluation of algorithmic outputs (31). In prosthodontic-related teaching, particularly in areas such as temporomandibular disorders and orofacial pain that rely heavily on structured history taking and differential diagnosis, ChatGPT-driven clinical case simulation has shown diagnostic reasoning performance comparable to that of real-patient simulation, highlighting its complementary value in the teaching of complex cases (32).

Furthermore, a large-scale international survey revealed that medical, dental, and veterinary students from 192 institutions in 48 countries generally held positive attitudes toward AI and expressed a clear demand for more AI-related education. Nevertheless, most participants considered current curricular provision inadequate and reported that they were not yet sufficiently prepared for the use of AI in their future professional practice. These findings, viewed from the demand side of education, further underscore the need for universities to accelerate the development of core AI curricula and tiered instructional strategies (33).

Taken together, AI-assisted tools appear to offer useful support for formative feedback, knowledge organization, and selected diagnostic or communication exercises. However, the strength of evidence varies considerably across applications (28–30, 32, 33). Studies evaluating narrowly defined and objectively measurable tasks, such as landmark identification or performance against reference answers, provide more direct evidence than surveys of student attitudes or uncontrolled classroom demonstrations. Nevertheless, even these studies rarely determine whether improved performance is retained over time or transferred to independent clinical and technical practice. Furthermore, favorable findings from endodontic, orthodontic, radiological, or general clinical education cannot be directly extrapolated to dental technology training, where competencies additionally include digital prosthesis design, manufacturing accuracy, quality control, and laboratory–clinical coordination.

Representative applications of artificial intelligence in dental education and the major limitations of the available evidence are summarized in Table 1.

## 5 Deep restructuring of practical teaching through progressive practice and the integration of virtual and real training

For dental technology programs, which place strong emphasis on technical application and engineering practice, the cultivation of core operational skills remains a key determinant of training quality. In recent years, the traditional one-way training model based primarily on typodonts and extracted teeth has gradually been supplemented by practical teaching approaches that place greater emphasis on project-based learning, the development of collaborative competence, and alignment with authentic workplace contexts. In the joint training of dentistry and dental technology students, project-based courses have been shown to enhance teamwork, independent thinking, and career-related competencies (34). At the same time, the deep integration of virtual reality (VR) and haptic feedback technologies has provided a repeatable, low-risk training environment with individualized feedback for preclinical skills development. Systematic reviews have shown that haptic simulators can significantly improve students' acquisition of psychomotor skills across multiple dental specialties and, to some extent, support more objective assessment based on differences in experience level, thereby providing an important technological foundation for restructuring practical teaching through the integration of virtual and real training (35).

### 5.1 Current evidence on haptics-enhanced virtual reality

Virtual reality and haptic systems provide repeatable, low-risk environments for psychomotor training and process-based feedback. Research activity in this area has increased rapidly, particularly in restorative and prosthodontic education (36). Systematic reviews suggest possible short-term improvements in operative performance and learner engagement, although the consistency and durability of these effects remain uncertain (35, 37). Simodont and similar platforms can simulate cutting sensations, three-dimensional visualization, and automated performance recording for procedures such as tooth preparation (38).

Individual studies have reported improvements in preparation time and selected morphological outcomes after haptic training (39). In endodontic education, combining virtual simulation, plastic teeth, and three-dimensional printed teeth was considered a useful staged approach, with virtual simulation viewed as most appropriate for introductory training (40). A systematic review and meta-analysis of prosthodontic education also suggested benefits for selected operative outcomes and task efficiency, although the findings were heterogeneous and mainly concerned short-term outcomes (41).

Overall, current evidence supports VR and haptic systems more clearly for early psychomotor training and immediate feedback than for long-term competency development. Differences in simulator type, training duration, comparators, assessment methods, and learner populations limit direct comparison across studies (35, 37). Most outcomes are measured during or immediately after training, and evidence concerning skill retention or transfer to authentic clinical and dental laboratory settings remains limited. Automated simulator scores may capture technical accuracy more readily than tactile judgment, material handling, communication, or context-dependent decision-making. These technologies should therefore complement rather than replace conventional practical training.

### 5.2 A “combination over replacement” strategy for integrating virtual and real training

Although the value of VR and haptic simulation in dental education has been widely recognized, current evidence generally supports their role as important complements to conventional physical training rather than as complete replacements (42). Regarding the overall design of simulation-based dental education, researchers have proposed the establishment of evidence-based best-practice frameworks and emphasized that simulation training should be systematically configured according to competency goals, training stage, and specific clinical tasks (42). In addition, a scoping review suggested that simulation-based dental education is not a single technological pathway, but rather requires the combination of different media and training contexts according to instructional objectives (43).

At the level of specific training tasks, a randomized controlled study showed that, in veneer tooth preparation training, VR simulators and conventional typodont-based training did not differ significantly in final learning outcomes. Thus, a more appropriate educational logic is not which method should replace the other, but how different training media can be combined according to instructional goals (44). This finding contrasts with studies reporting improvements in preparation scores or task-completion efficiency following VR-based training (39, 41). The inconsistency may reflect differences in task complexity, baseline learner experience, training duration, feedback design, and outcome assessment. Therefore, the current evidence does not establish the universal superiority of virtual simulation over conventional training; rather, the educational value of each modality is likely to depend on the specific learning objective and stage of training. For tasks involving complex anatomical cognition, extended reality (XR) and augmented reality (AR) technologies may offer specific educational advantages. In the teaching of root canal anatomy, for example, augmented reality-based instruction significantly improved students' accuracy in anatomical identification and reduced learning time, making it more suitable as a tool for spatial cognition and early conceptual understanding (45).

During the transition toward real clinical procedures, virtual models and 3D-printed models derived from real patient data demonstrate greater simulation fidelity. Studies have shown that both students and instructors consider patient-specific virtual models and 3D-printed tooth models helpful for improving confidence, autonomy, and clinical readiness in preclinical training (46). Another review further noted that, because of their good standardization, accessibility, and anatomical realism, 3D-printed teeth can serve as important supplements to extracted teeth and conventional standard models, while also improving learning experience, clinical skills, and learner confidence (47).

For dental technology education, the value of digital training also lies in its ability to advance workplace competency development. A prospective cohort study showed that a virtual 3D simulation-based digital training module improved long-term competency retention and training efficiency in removable partial denture design. Such training is therefore more suitable for the middle and later stages of integrated practice, where it can work together with physical fabrication and real-case communication to form a complete workflow-based training model (48).

Therefore, in reforming practical teaching, application-oriented undergraduate universities should adopt a strategy of integrating virtual and real training, with combination taking priority over replacement. VR and haptic simulation can be used for low-risk trial-and-error learning and foundational skill acquisition; XR can support spatial understanding of complex structures; patient-specific 3D-printed models can facilitate realistic preclinical transition; and digital design training can then be extended into authentic workflow settings. Together, these elements can form a progressive practical teaching pathway linking cognition, simulation, realistic rehearsal, and workplace application. The educational value, evidence limitations, and recommended roles of emerging technologies in dental training are critically summarized in Table 2.

## 6 Comprehensive curriculum restructuring through digital workflows and blended learning

For students in dental technology programs, mastery of the digital workflow has become an essential component of core professional competence. In recent years, the integration of online and blended learning models has begun to break the traditional constraints of time and space in teaching, enabling digital theories and operational procedures to be incorporated into the curriculum more efficiently (49).

### 6.1 Full-cycle integration of the digital workflow

Studies on the implementation of preclinical courses have shown that digital dental technologies are being progressively incorporated into undergraduate education, with generally positive student perceptions. However, their educational effectiveness still depends substantially on equipment availability, curriculum design, and the quality of instructor feedback (50). In areas such as removable partial dentures, complete dentures, and fixed prosthodontics, traditional clinical and laboratory workflows are increasingly being restructured through intraoral scanning, CAD/CAM design, and digital analysis tools. Survey data indicate that digital removable prosthodontic content has already been incorporated into the curricula of many dental schools, although its broader adoption remains constrained by factors such as cost, software platforms, information technology support, and faculty training (51). In addition, the use of intraoral scanners as self-directed learning tools in tooth preparation training has shown considerable educational potential. Studies have demonstrated that combining hands-on operation with immediate evaluation using digital analysis tools can improve students' operative performance and provide more objective feedback, and undergraduate students generally hold positive attitudes toward the integration of such digital self-assessment tools into preclinical teaching (52).

However, current evidence on digital workflow education is derived largely from institutional surveys, student perceptions, and short-term preclinical performance assessments (50–52). Positive attitudes toward intraoral scanning or CAD/CAM training, and even improvements in isolated technical tasks, do not necessarily demonstrate that students can independently complete an entire digital prosthetic workflow or transfer these skills to workplace settings. The applicability of findings from institutions with established digital resources to application-oriented universities with more limited infrastructure also remains uncertain. In addition, evidence regarding full-workflow competence, cross-platform adaptability, cost-effectiveness, software obsolescence, and graduate workplace performance remains insufficient. Curriculum development should therefore emphasize transferable workflow understanding and quality-control competence rather than proficiency in a single device or software platform.

Direct evidence specific to dental technology remains limited. Existing studies have examined the integration of digital technologies into dental technician education and students' perceptions and educational needs regarding digital curricula (5, 53, 54). A prospective cohort study involving dental technology students reported improved and retained performance in removable partial denture design following virtual 3D training (48), while a pilot study explored the use of three-dimensional anthropomorphic models in a dental technician curriculum (55). Although these findings support the relevance of digital design and workflow-based training, evidence concerning CAD/CAM technologist training, complete prosthesis fabrication workflows, manufacturing quality control, and clinical–laboratory integration remains sparse. Findings from general dental or medical education should therefore be regarded as indirect evidence and should not be assumed to transfer automatically to laboratory-based dental technology training.

### 6.2 Digital empowerment of basic medical education and communication skills training

Beyond engineering and technical training, digital intelligence-driven reform is also reshaping the cultivation of foundational knowledge and communication skills. In basic courses such as tooth morphology, digital learning modules that incorporate online three-dimensional tooth models, discussion-based teaching, and digital quizzes can enhance students' understanding of dental three-dimensional morphology and improve both learning outcomes and course experience (56). In addition, in the teaching of clinical communication and behavioral skills, researchers have developed digital game-based systems for dental students. Through role-playing and branching scenario simulations, these systems help students understand dentist–patient communication contexts in interactive environments and improve both their learning motivation and communication-related self-efficacy (57). To further summarize the relationship between digital curriculum development and professional competency cultivation, the major course modules, relevant technologies, and competency goals in dental technology education are outlined in Table 3.

**Table 3:** Digital curriculum modules and competency alignment.

Course module	Main technology	Core outcome	References
Dental anatomy	3D models; XR/AR	Spatial understanding	(45, 56)
Fixed prosthodontics	IOS; CAD/CAM; VR	Design and preparation	(38, 41, 52)
RPD design	Virtual 3D software	Design and problem-solving	(48)
Complete dentures	Digital setup; online learning	Workflow and self-learning	(49, 61)
Digital prosthodontics	CAD; 3D printing	Workflow integration	(48–52)
Imaging and data	CBCT; AI analysis	Image and data interpretation	(29–31)
Communication	LLMs; virtual patients	Communication and reasoning	(24–27, 57)
Practice and internship	Digital workflows; 3D models	Workplace transfer	(34, 48, 55)

AI, artificial intelligence; AR, augmented reality; CAD/CAM, computer-aided design and computer-aided manufacturing; CBCT, cone-beam computed tomography; IOS, intraoral scanning; LLMs, large language models; RPD, removable partial denture; VR, virtual reality; XR, extended reality.

## 7 Constraints and governance pathways for digital intelligence-driven reform in application-oriented undergraduate contexts

Although international research continues to advance the digital transformation of dental education, such reforms cannot simply replicate the models used by research-intensive universities when applied to application-oriented undergraduate institutions. A study on students' perceptions of digital dental technology curricula showed that, although most students recognized the value of these courses for professional learning, career choice, and practical work, a clear gap still exists between course content and workplace practice requirements. This finding suggests that, in the process of digital intelligence-driven transformation, application-oriented talent cultivation continues to face practical constraints related to insufficient alignment between curriculum development and competency demands (53).

### 7.1 Resource barriers and the curriculum-workplace gap

Studies involving dental technology students have shown that although most learners recognize the importance of digital courses for professional learning, career choice, and practical work, a considerable proportion still lack confidence in independently completing digital design tasks after completing these courses. In addition, the proportion of graduates entering relevant digital positions remains lower than expected, indicating a persistent gap between curricular content and workplace practice (53). At the same time, reviews of digital curriculum implementation have pointed out that equipment procurement, software licensing, maintenance and upgrading, information infrastructure, and faculty training all generate ongoing cost pressures. These factors are also important reasons why many institutions find it difficult to achieve high-density equipment deployment and high-frequency practical training (54). Against this background, some institutions have begun to incorporate 3D-printed anthropomorphic models into dental technology curricula. Pilot studies have shown that such models are well accepted by students, help strengthen interest in digital technologies and confidence in related skills, and may serve as a more feasible transitional teaching resource alongside high-cost full-process equipment (55).

Taken together, the available evidence reveals an important discrepancy between perceived educational value and demonstrated occupational readiness. Although students may recognize the relevance of digital courses, some remain insufficiently confident in independently completing digital design tasks, and the proportion entering related digital positions remains lower than expected (53). This discrepancy indicates that student satisfaction, perceived usefulness, and self-reported confidence should not be treated as equivalent to demonstrated professional competence. Similarly, the favorable acceptance of lower-cost teaching resources, such as three-dimensional printed models, does not by itself establish long-term technical proficiency or cost-effectiveness (55). Future evaluations should therefore incorporate objective full-workflow performance, quality-control outcomes, internship assessment, and graduate workplace follow-up.

Cost-effectiveness remains insufficiently evaluated because most studies report educational outcomes without considering equipment, software, maintenance, staffing, technical support, and opportunity costs (13, 49, 51). Implementation feasibility also varies across institutions: well-funded programs may support integrated platforms and frequent student access, whereas under-resourced settings may require phased adoption, shared facilities, lower-cost technologies, or industry partnerships (7, 13). Without context-sensitive strategies, digital reform may widen disparities in educational opportunities.

### 7.2 Institutional, curricular, and governance barriers to implementation

Beyond financial and technological constraints, implementation also depends on faculty engagement and curriculum capacity. Faculty resistance may arise from unfamiliarity with new technologies, increased workload, uncertainty about changing professional roles, or doubts regarding the educational value of AI-assisted tools. Adding AI, CAD/CAM, virtual simulation, and data-literacy content to an already crowded curriculum may also result in fragmented learning. Institutions should therefore use curriculum mapping, consolidate redundant content, provide faculty-development support, and align new digital competencies with institutional outcomes and accreditation requirements (13).

The use of cloud-based platforms, patient records, intraoral scans, radiographic data, learning analytics, and generative AI raises concerns regarding cybersecurity, data privacy, intellectual property, and accountability. Institutional policies should define approved platforms, data de-identification and access control, secure storage and transmission, ownership of digital and AI-generated materials, permitted uses of AI, disclosure requirements, human verification, and responsibility for erroneous or biased outputs (21, 22).

Assessment validity and institutional readiness also require careful consideration. Automated scoring and AI-generated feedback may improve efficiency, but model-dependent grading differences and the potential over-scoring of incorrect responses indicate that such tools should not be used for high-stakes decisions without faculty oversight (58). Before large-scale implementation, institutions should assess leadership commitment, financial sustainability, infrastructure, faculty digital competence, curriculum capacity, information-technology and cybersecurity support, and student readiness (13, 22). Faculty development should include the operation of digital systems, critical interpretation and verification of AI-generated outputs, data protection, and the integration of digital tools into teaching and assessment, while students may require staged orientation and additional practice to accommodate different learning curves and levels of prior digital experience. A practical implementation pathway should progress from readiness assessment and competency mapping to small-scale piloting, phased expansion, and continuous evaluation of access, utilization, learning outcomes, maintenance burden, cost, and workplace feedback (13, 50).

The financial, curricular, institutional, technological, assessment-related, and governance barriers to digital intelligence-driven reform, together with corresponding implementation strategies, are summarized in Table 4.

**Table 4:** Key barriers and implementation strategies.

Challenge	Main issue	Educational impact	Suggested strategy
Cost and infrastructure	High purchase and maintenance costs	Limited access	Phased investment; shared resources(13, 49, 51)
Faculty and curriculum	Skill gaps; resistance; overload	Uneven teaching	Training; curriculum mapping (13, 59, 63)
Accreditation	Poor standards alignment	Unclear outcomes	Map competencies to standards (12, 16, 59)
Workplace gap	Few authentic workflows	Weak skill transfer	Cases; projects; internships (34, 53, 59)
Data and privacy	Patient, learner, and cloud-data risks	Breach or misuse	Approved systems; access control; de-identification (21, 22, 63)
AI governance and IP	Bias, hallucination, unclear ownership	Integrity and decision risks	Disclosure; ownership rules; human review (20–22, 63)
Assessment validity	Unvalidated AI scoring	Incomplete or biased grading	Tool validation; faculty oversight (58)
Institutional readiness	Weak leadership, IT, or funding	Poor sustainability	Readiness review; pilot; phased rollout (7, 13, 22)

AI, artificial intelligence; IP, intellectual property; IT, information technology. Strategies are context-dependent recommendations rather than validated universal interventions.

### 7.3 A literature-informed integrative conceptual framework for curriculum planning: three-stage practice, three-dimensional optimization, and dual integration

The framework proposed in this review is a literature-informed integrative conceptual framework newly synthesized by the authors for application-oriented dental technology education. It is not intended to replace established competency-based education models or to represent a newly validated pedagogical theory. Its originality lies in translating broad competency-based principles into a context-specific structure that simultaneously links the progression of practical training, the coordinated optimization of curriculum implementation, and collaboration across disciplinary and institutional boundaries.

Within this framework, “three-stage practice” refers to a progressive pathway from(1) virtual and simulation-based foundational learning, to(2) physical laboratory and case-based integrated training, and finally to(3) clinical observation, enterprise internship, and workplace application. “Three-dimensional optimization” refers to the coordinated optimization of curriculum content, teaching and learning processes, and assessment and feedback. “Dual integration” refers to medicine–engineering integration and industry–education integration. Existing competency-based educational models primarily define the professional outcomes and capabilities that learners are expected to achieve. The present framework extends this logic by specifying how these competencies may be progressively developed, assessed, and transferred across virtual, physical, clinical, and workplace settings in dental technology programs.

Within this framework, competency assessment should combine objective task performance, digital portfolio evidence, and longitudinal workplace evaluation. Technical competence may be assessed using standardized full-workflow tasks, analytic scoring rubrics, the accuracy of digital designs and fabricated prostheses, completion time, error or remake rates, and independent quality-control checks. Digital competence should include data acquisition, software operation, workflow integration, troubleshooting, data security, and cross-platform adaptability. AI literacy may be evaluated through knowledge tests and scenario-based tasks addressing source verification, hallucination and bias recognition, explainability, ethical use, and appropriate human oversight (12, 16, 58). To evaluate transfer beyond simulation, student performance should be compared across virtual training, physical laboratory tasks, internships, and authentic cases. Graduate outcomes may be followed through repeated competency assessments, workplace performance indicators, and employer feedback (48, 50).

The rationale for the individual components of the framework is supported by several strands of existing evidence. Digital health competency frameworks support the reconstruction of learning outcomes around digital literacy, professional judgment, ethical awareness, and interdisciplinary collaboration (12, 15, 16). Studies of virtual reality, haptic simulation, three-dimensional printed models, and digital design training support a staged combination of virtual, physical, and workplace-oriented learning rather than the replacement of conventional practical training (39–45). Evidence relating to digital curricula, interprofessional education, online learning, and AI-assisted feedback further supports curriculum alignment, collaborative learning, and formative assessment (50, 59–62). However, these studies evaluate separate educational components rather than the integrated framework as a whole.

Accordingly, the proposed framework should be interpreted as a conceptual guide for curriculum planning and local implementation rather than as an intervention with established effectiveness. Although the available literature supports the educational rationale for its individual components, no prospective study has yet evaluated the combined framework in its entirety. Its feasibility, effectiveness, and transferability should therefore be examined through longitudinal and multicenter studies using objective competency measures, internship performance, graduate workplace outcomes, and comparisons across institutions with different levels of resources.

## 8 Discussion

Digital intelligence-driven reform in dental technology education should be understood not merely as the adoption of isolated digital tools, but as a systematic reconstruction of curriculum objectives, competency frameworks, teaching processes, assessment methods, and governance arrangements. Digital competence should include not only proficiency in intraoral scanning, CAD/CAM, three-dimensional printing, and AI-assisted tools, but also data literacy, algorithmic awareness, critical appraisal, digital professionalism, ethical judgment, and interdisciplinary communication (12). Although large language models may support personalized learning, case-based teaching, content generation, and formative feedback, these benefits should not obscure the risks of hallucinated information and fabricated references (17, 26). Algorithmic bias and limited explainability may further reduce the fairness, transparency, and trustworthiness of AI-generated educational outputs (63).

Reproducibility is also uncertain because AI outputs may change with model updates, prompt wording, input context, or generation settings (19). These limitations are particularly important for novice students, who may lack sufficient knowledge to identify plausible but inaccurate outputs and may become overly dependent on AI for information retrieval and reasoning (18). AI-generated teaching content, feedback, and assessment results should therefore be validated by qualified faculty and should not serve as the sole basis for high-stakes assessment or professional decisions. The reported views of dental educators also indicate the need for faculty preparedness, structured guidance, and clear institutional boundaries for AI use (62). Because regulatory requirements remain uncertain and continue to evolve, institutions should establish policies defining permitted uses, disclosure requirements, human oversight, accountability, and periodic review (21, 22).

Another key issue concerns the relationship between virtual simulation and conventional hands-on training. Virtual reality and haptic simulators can provide repeatable, measurable, and low-risk training environments, especially for early-stage psychomotor skill acquisition and process-based feedback (35, 64). However, current evidence does not support the complete replacement of conventional laboratory training or typodont-based practice by virtual simulation alone (42, 44). Instead, different digital tools should be integrated according to specific learning objectives and training stages. For example, XR/AR technologies may be particularly useful for anatomical cognition and spatial understanding, while patient-specific 3D-printed models may help students transition from standardized preclinical exercises to more realistic clinical scenarios (45). In addition, digital design platforms may strengthen workflow-based competence in the middle and later stages of practical training, particularly when combined with physical fabrication and real-case communication (48). Thus, the core of practical teaching reform is not a simple choice between virtual or real training, but the construction of a progressive pathway linking cognition, simulation, physical operation, clinical observation, and workplace practice.

Across technologies, the maturity of the available evidence differs substantially. Relatively stronger evidence supports virtual reality and haptic simulation for short-term psychomotor training and immediate formative feedback, although findings remain heterogeneous and do not establish consistent superiority over conventional training (35, 41, 44). One prospective cohort study also suggested that digital simulation may support longer-term retention in removable partial denture design, although transfer to workplace performance was not evaluated (48). By contrast, evidence concerning generative AI and automated assessment remains preliminary and is based mainly on pilot studies, single-center evaluations, and review-level synthesis (17, 58, 65). Evidence for XR and AR is also limited and largely task-specific (45). Across these technologies, evidence concerning sustained workplace outcomes remains insufficient.

Taken together, the current evidence base remains uneven and does not support firm conclusions regarding sustained or generalizable educational effectiveness. Much of the literature consists of pilot studies, single-center educational simulations, and short-term pre–post evaluations (27, 54), whereas broader conclusions often rely on cross-sectional surveys and narrative or scoping reviews (34, 65). These designs are useful for assessing feasibility, learner acceptance, and immediate performance, but they cannot establish causality, long-term competency development, or transfer to authentic workplace practice. Only a limited number of studies have examined retention beyond the immediate training period; for example, one prospective cohort study followed dental technology students for up to 1 year (48). Multicenter studies evaluating sustained educational benefit, workplace performance, and graduate outcomes remain rare. Considerable heterogeneity in learner populations, technologies, comparators, training duration, and outcome measures further limits direct comparison.

Evidence involving dental technology students is especially limited, and findings from general dental or medical education may not be directly transferable to laboratory-based professional training. Unlike general competency-based frameworks that primarily define expected educational outcomes, the proposed framework provides a context-specific pathway for developing and assessing competencies across virtual, physical, clinical, and workplace settings. Its originality lies in integrating staged practical training, coordinated optimization of curriculum content, teaching processes, and assessment, together with medicine–engineering and industry–education collaboration. Nevertheless, it should be interpreted as a literature-informed conceptual framework rather than as a validated intervention with established effectiveness.

### 8.1 Limitations

This review has several limitations. First, although a structured search strategy and explicit eligibility criteria were used, it was conducted as a narrative rather than a systematic review. No protocol was prospectively registered, and no formal risk-of-bias assessment or quantitative meta-analysis was performed; therefore, incomplete retrieval, publication bias, and some subjectivity in the selection of representative literature cannot be excluded. Second, much of the available evidence is derived from pilot studies, single-center evaluations, cross-sectional surveys, and short-term educational outcomes, limiting conclusions regarding sustained competency development and workplace transfer. Third, direct evidence involving dental technology students remains limited, particularly in relation to CAD/CAM training, removable prosthodontic workflows, intelligent prosthesis manufacturing, quality control, and clinical–laboratory integration. Findings from general dental or medical education should therefore be interpreted cautiously when applied to dental technology programs. Finally, AI models, software platforms, access conditions, and regulatory guidance are evolving rapidly, which may affect the continuing applicability of the evidence and recommendations summarized in this review.

These limitations highlight the need for prospective, multicenter, and longitudinal studies using objective competency measures, standardized comparators, internship assessment, workplace performance, and graduate follow-up. Future research should give particular attention to dental technology-specific outcomes, including digital prosthesis design, complete manufacturing workflows, quality control, and clinical–laboratory collaboration. The feasibility, cost-effectiveness, and educational value of integrated digital workflows, AI governance, medicine–engineering collaboration, and industry–education synergy should also be evaluated across institutions with different levels of resources.

## 9 Conclusion

In conclusion, digital intelligence is reshaping the competencies required in dental technology education and provides opportunities to extend conventional training through digital workflows, virtual simulation, three-dimensional printing, and artificial intelligence-assisted learning. However, these technologies should be integrated as supervised complements to conventional laboratory training, faculty instruction, and authentic clinical or workplace experience rather than as substitutes for them. Implementation should therefore include faculty validation, transparent disclosure, and periodic governance review to mitigate AI-related risks and regulatory uncertainty. The literature-informed conceptual framework proposed in this review integrates three-stage practice—from virtual and simulation-based learning to physical laboratory training and clinical or workplace application—with three-dimensional optimization of curriculum content, teaching and learning processes, and assessment and feedback, as well as the dual integration of medicine with engineering and education with industry. Its originality lies in the context-specific integration of these established educational principles for application-oriented dental technology programs, rather than in the creation of a new competency-based educational theory. Although existing studies support the rationale for individual components of the framework, the framework as a whole has not yet been empirically validated. It should therefore be regarded as a conceptual guide for curriculum planning that requires adaptation to institutional resources, faculty capabilities, and local workforce needs. Future multicenter and longitudinal studies should evaluate its feasibility, cost-effectiveness, effects on objectively measured competence, transfer to workplace performance, and sustained graduate outcomes.
